# Supplementary material for: Degradation and Pathways of Carvone in Soil and Water
Source: Molecules. 2022 Apr 8;27(8):2415. doi: 10.3390/molecules27082415 (PMC9027270; doi:10.3390/molecules27082415)
Supplement: Supplementary file 1 [file molecules-27-02415-s001.zip › Supplementary Material..pdf]

Degradation and Pathways of Carvone in Soil and Water

Chenyu Huang [1], Wenwen Zhou [2], Chuanfei Bian [1], Long Wang [1], Yuqi Li [3] and Baotong Li [1,*]

[1] College of Land Resources and Environment, Jiangxi Agricultural University, 330045, Nanchang, China; ChenyuHuang999@163.com (C.H.); bcf940331@163.com (C.B.); WL2283807483@163.com (L.W.)
[2] College of Food Sciences, Jiangxi Agricultural University, Nanchang 330045, China; fly_zww@163.com
[3] College of Engineering, Jiangxi Agricultural University, Nanchang 330045, China; tonglm66@163.com
* Correspondence: libt666@163.com; Tel: +86-15179409965

Table S1. Linear equations, R^2, matrix effects and limits of quantification of carvone and its degradations in n-hexane, soil and aqueous solutions.

Compound	Matrix	Regression equation	R^2	Matrix effect	LOQ (μg kg^{-1})
(-)-Carvone	N-hexane	y=20103.3x+59.0	0.9998	-	10
	S1	y=17690.6 x +61.7	0.9994	-0.12	10
	S2	y=22185.4x+41.4	0.9994	0.10	10
	S3	y=19700.9 x +48.5	0.9997	-0.05	10
	S4	y=19098.1x+65.5	0.9996	-0.05	10
	PH=4	y=19902.3x+55.3	0.9998	-0.01	10
	PH=7	y=20304.3x+66.9	0.9998	0.01	10
	PH=9	y=20505.4x+53.8	0.9998	0.02	10
Carvonecamphor	N-hexane	y=62691.2x+788.0	0.9998	-	30
	PH=7	y=62565.8x+754.3	0.9998	-0.02	30
Dihydrocarveol	N-hexane	y=10486.3x-21.9	0.9998	-	10
	S2	y=10099.7x-91.8	0.9978	-0.04	10
Dihydrocarvone	N-hexane	y=2170.6x+9.6	0.9999	-	50
	S2	y=2104.1x+38.9	0.9978	-0.03	50

Table S2. Average recoveries and RSDs (n=5, %) for target compounds from different matrices at three spiked levels.

Matrix	Spiked level (μg kg^{-1})	(-)-carvone		Dihydrocarveol		Dihydrocarvone		Carvone camphor	
		Recovery (%)	RSD (%)	Recovery (%)	RSD (%)	Recovery (%)	RSD (%)	Recovery (%)	RSD (%)
S1	50	91.2	2.5	82.0	5.8	93.3	8.4		
	500	92.1	3.7	77.7	6.4	84.3	4.7		
	5000	99.2	9.6	80.5	4.8	88.8	5.6		
S2	50	86.1	4.3	82.7	5.1	85.6	9.7		
	500	80.5	5.4	80.4	6.4	87.6	5.8		
	5000	85.4	7.6	79.5	5.4	84.3	4.3		
S3	50	78.5	2.9	71.9	11.3	82.5	5.4		
	500	85.4	6.2	88.4	5.7	93.4	3.3		
	5000	75.8	3.7	83.0	5.9	95.4	2.8		
S4	50	90.09	3.1	89.1	2.3	89.4	2.1		
	500	99.89	1.5	77.4	1.1	92.5	1.9		
	5000	96.33	4.7	81.8	5.5	84.3	2.8		
pH=4	50	99.12	2.2					93.51	3.7
	500	98.5	1.8					90.41	4.3
	5000	97.81	2.4					96.57	2.8
pH =7	50	91.55	6.9					98.83	1.8
	500	94.97	1.5					99.92	2.4
	5000	94.82	1.7					91.76	2.8
pH =9	50	95.84	2.2					90.95	2.3
	500	95.59	1.4					97.43	2.2
	5000	98.7	1.8					96.95	4.5
